# What is the identity of Gerota fascia? Histological study with cadavers

**DOI:** 10.1111/iju.15596

**Published:** 2024-10-22

**Authors:** Yasuyuki Kobayashi, Kohei Edamura, Takuya Sadahira, Yusuke Tominaga, Satoshi Katayama, Takehiro Iwata, Shingo Nishimura, Tomoko Kobayashi, Keita Sato, Takaaki Komiyama, Ryusuke Momota, Hideyo Ohuchi, Motoo Araki

**Affiliations:** ^1^ Minimally Invasive Therapy Center Okayama University Hospital Okayama Japan; ^2^ Department of Urology Okayama University Hospital Okayama Japan; ^3^ Organ Transplant Center Okayama University Hospital Okayama Japan; ^4^ Department of Cytology and Histology Okayama University Graduate School of Medicine, Dentistry and Pharmaceutical Sciences Okayama Japan; ^5^ Department of Human Morphology Okayama University Graduate School of Medicine, Dentistry and Pharmaceutical Sciences Okayama Japan; ^6^ Department of Urology Okayama University Graduate School of Medicine, Dentistry and Pharmaceutical Sciences Okayama Japan

**Keywords:** collagen fiber, connective tissue, fusion fascia, Gerota fascia, renal fascia

## Abstract

**Objectives:**

The advancement of laparoscopic surgery has allowed surgeons to see finer anatomical structures during surgery. As a result, several issues have arisen regarding Gerota fascia that cannot be explained by previous interpretations, such as its various forms observed during surgery. To address these issues, we histologically examined the structure of Gerota fascia.

**Methods:**

Specimens for study were prepared from kidneys with Gerota fascia from four cadavers, and the structure was studied histologically. Its thickness and collagen fiber area ratios were measured using ImageJ and compared to those of the epimysium of the rectus abdominis muscle.

**Results:**

Connective tissue that appeared to be Gerota fascia was observed in 26 specimens. Histologically, the basic structure of Gerota fascia was a sandwich‐like structure with a thin layer of thick, long collagen fibers in the central layer, and small granular collagen fibers scattered at the edges. However, not all areas observed had a similar structure; eight specimens were composed only of small granular collagen fibers. The average thickness of the Gerota fascia was 466 μm, and the area ratio of collagen was 27.1%. In contrast, the epimysium was much thicker than Gerota fascia, and its collagen fibers were much thicker and denser.

**Conclusions:**

Gerota fascia, unlike the epimysium, was a very thin and fragile layer of collagen fibers, and its structure was diverse. This explains why Gerota fascia was observed in various states during surgery. It is important for surgeons to understand the properties of Gerota fascia and to treat it appropriately.

**Abbreviations & Acronyms**
ARCF: the area ratio of collagen fibers
EVG: Elastica van Gieson
Gf: Gerota fascia
HE: hematoxylin–eosin
MA: Masson trichrome

## INTRODUCTION

With advances in endoscopic technology, the anatomical structures that surgeons see are becoming finer. One example is Gerota fascia (Gf), which allows us to see finer structures than ever before, which has caused controversy over how Gf is viewed and handled. Gf observed in a transabdominal approach is often easily recognized as a distinct membrane. In contrast, such membranous structures are often not recognizable in the retroperitoneal approach. Even with the transabdominal approach, some cases are not distinct membranes and can only be recognized as loose connective tissue, and in the same case, some areas can be easily identified as membrane while others cannot. Why do these differences occur even though they are the same Gf? It is difficult to observe the original in vivo state of Gf because connective tissue such as Gf easily undergoes structural changes when external force is applied. Therefore, in this study, Gf specimens were collected from cadavers without detaching the surrounding organs to prevent Gf from being changed by external forces, and Gf was observed histologically to determine the true nature of Gf.

## MATERIALS AND METHODS

Gf is also called renal fascia, and in a broad sense, it may generally refer to both the anterior and posterior lobes of renal fascia, but in this study, the anterior lobe of renal fascia was defined as Gf.

**FIGURE 1:**
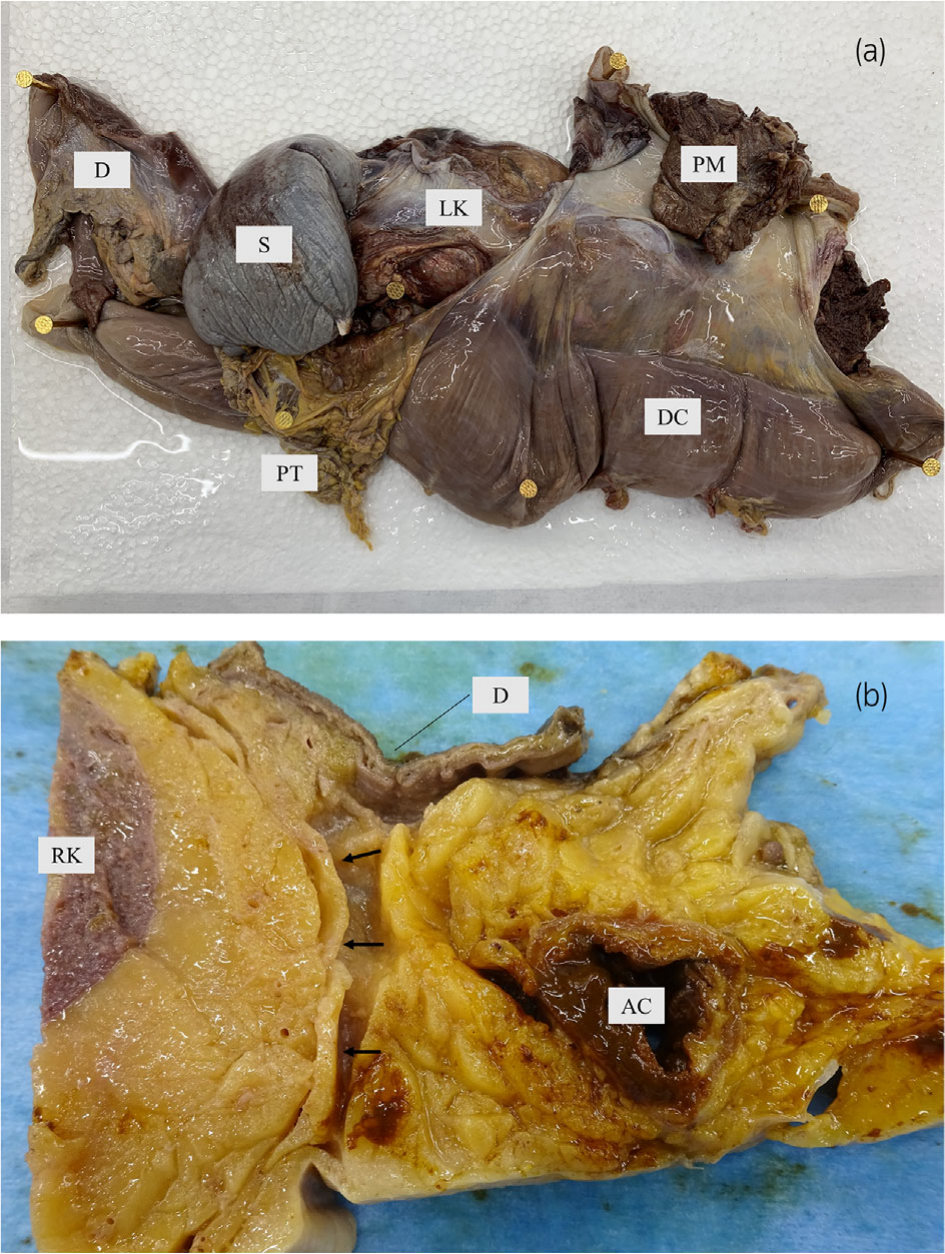
(a) Removal of the left kidney: The peritoneum, diaphragm, descending colon and its mesentery, pancreatic tail, spleen, and left adrenal gland were removed as a mass without any dissection of Gerota fascia layer (Cadaver No. 1). D, diaphragm; DC, descending colon; LK, left kidney; PM, psoas muscle; PT, pancreatic tail; S, spleen. (b) Cross sectioning of the right kidney: the duodenum and descending colon were cut out attached to the kidney without any dissection of Gf (Cadaver No. 1). AC, ascending colon; D, duodenum; RK, right kidney; arrows, Gerota fascia.

Four cadavers were used in this study. The details are shown in Table 1. To prevent breakage of the histological structure of Gf during removal of the kidney, the kidney was removed without detaching the layers of Gf and with the surrounding organs of the kidney attached. Specifically, in the case of the left kidney, part of the peritoneum, descending colon, mesentery, pancreatic tail, diaphragm, psoas muscle, spleen, and left adrenal gland were removed together (Figure 1a). The right kidney was removed along with part of the peritoneum, ascending colon, duodenum, psoas muscle, inferior vena cava, liver, and right adrenal gland. The kidneys and organs were fixed for 7 days using a 10% neutral buffered formalin solution and then excised for observation. When excising the specimens, the kidney and the surrounding organs were kept attached so that as little external force as possible was applied to the Gf layer (Figure 1b). An adequate specimen was defined as one in which Gf layer was undamaged and the surrounding organs remained attached. They were embedded in paraffin, cut in 5 μm sections, and subsequently stained with hematoxylin–eosin (HE), Masson trichrome (MA), and Elastica van Gieson (EVG). Histological studies were then performed on them.^1^ The thickness and the area ratio of collagen fibers (ARCF) of Gf were measured using ImageJ and compared to the control, the epimysium of the rectus abdominis muscle of cadaver No. 1.^2^ The ARCF was calculated after binarization using ImageJ.^2^ ImageJ is an open source image processing and analysis software developed by the National Institutes of Health (NIH). All statistical analyses were performed using JMP (version 5; SAS Institute, North Carolina, USA) with the Dunnett's test. *p* < 0.05 was considered significant.

**TABLE 1:**
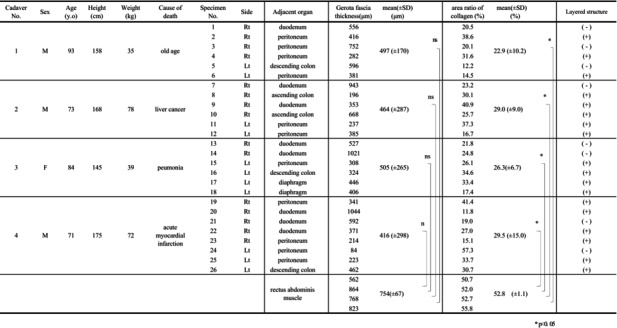
The details of the 4 cadavers and histological findings.

* *p* < 0.05.

## RESULTS

**FIGURE 2:**
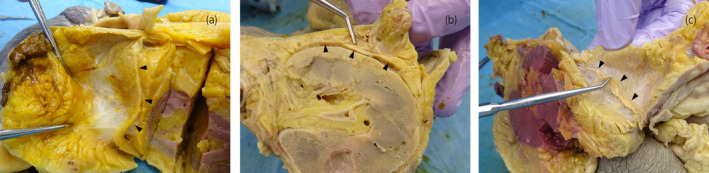
Grossly observed Gf (a) between left kidney and descending colon in Cadaver No. 2, (b) between left kidney and descending colon in Cadaver No. 3, (c) between left kidney and descending colon in Cadaver No. 4, arrowheads, Gerota fascia.

**FIGURE 3:**
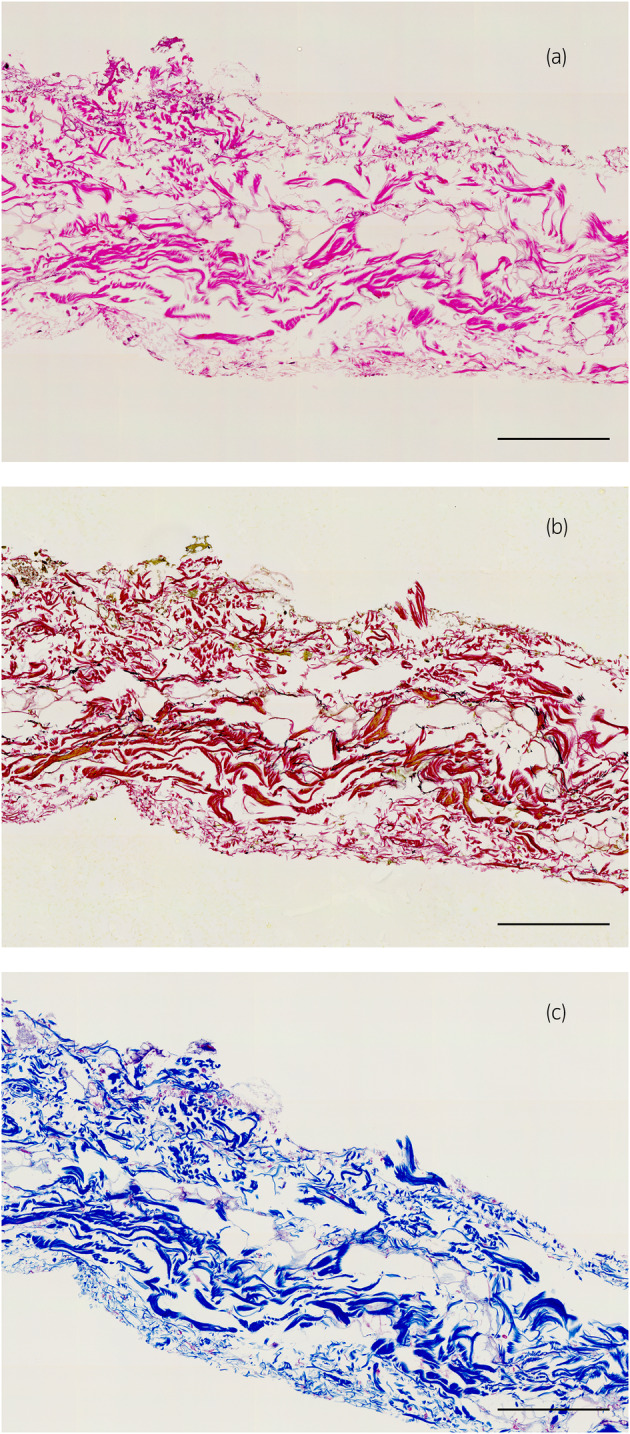
Photomicrographs of specimen 26 prepared with Gerota fascia only, (a) HE stain, (b) EVG stain, (c) MA stain. Scale bars: 200 μm (a–c).

**FIGURE 4:**
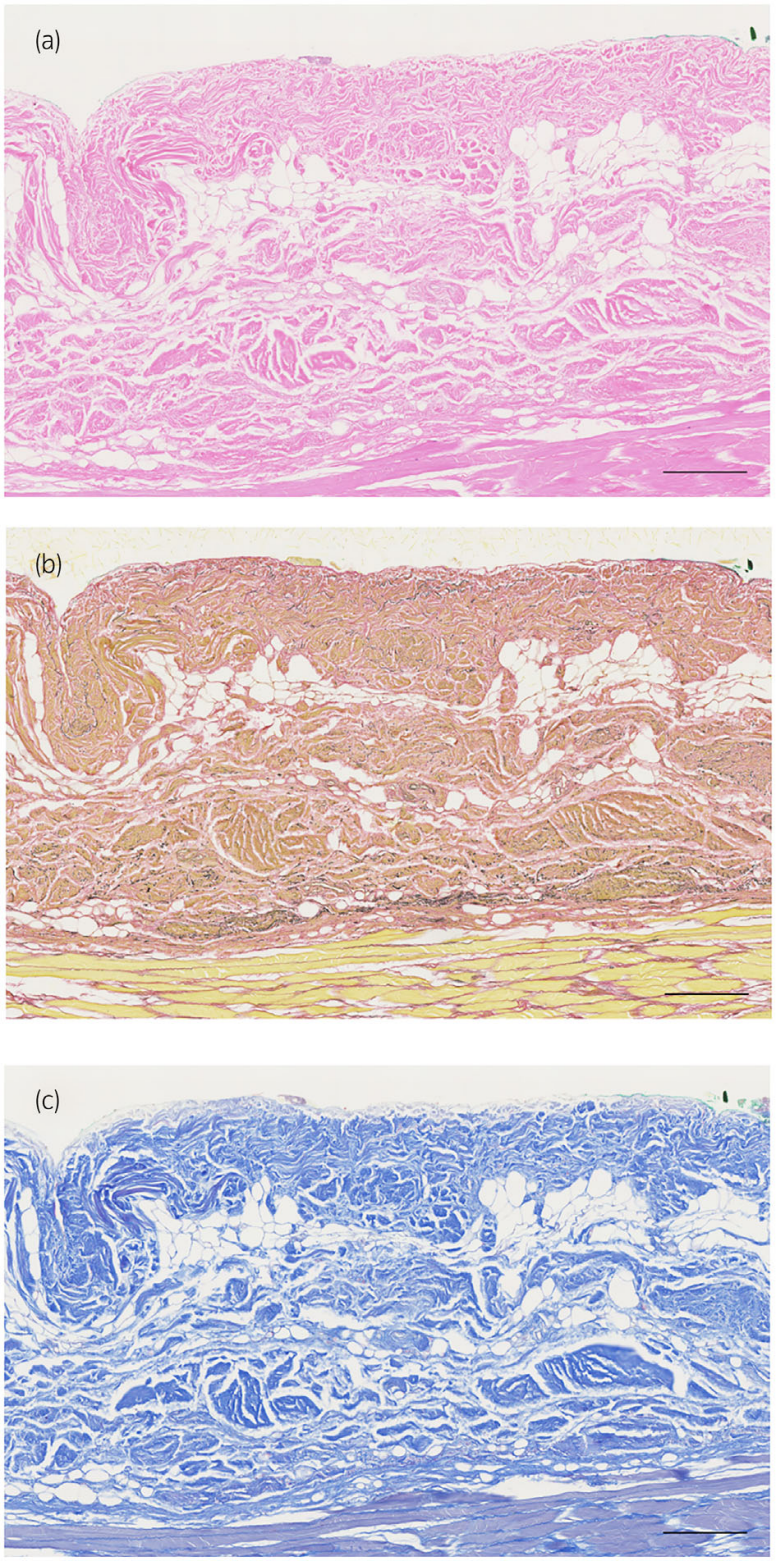
Photomicrographs of the outer membrane of the rectus abdominis muscle (a) HE stain, (b) EVG stain, (c) MA stain. Scale bars: 200 μm (a–c).

**FIGURE 5:**
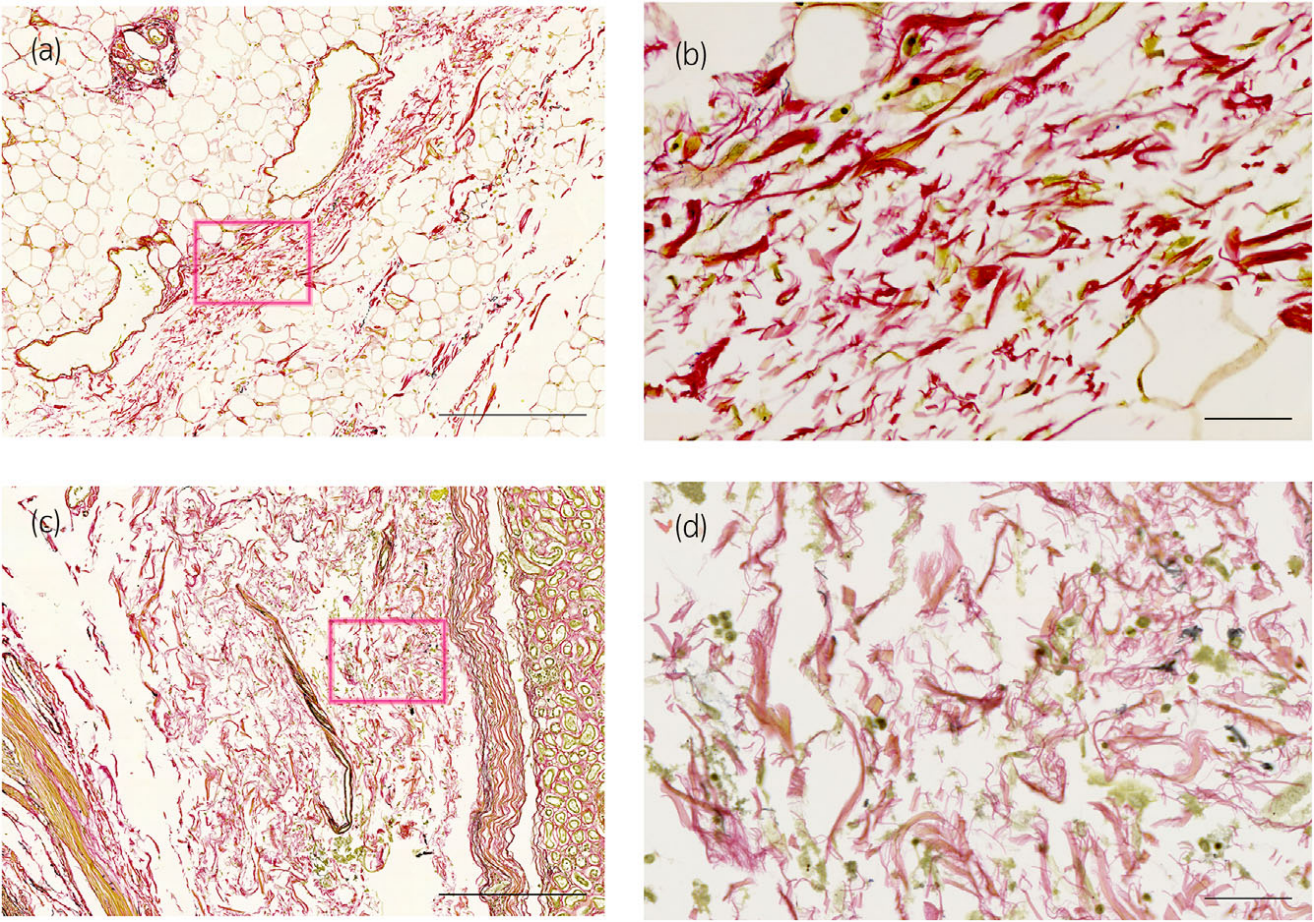
EVG stain. Microscopic findings of Gf lacking layer structure. Boxed areas in (a, c) are enlarged in (b, d), respectively. (a, b) Gf of specimen No. 7: no obvious layered structure, composed of small granular collagen fibers. (c, d) Gf of specimen No. 14: no obvious layered structure, composed of small granular collagen fibers and short non‐directional collagen fibers. Scale bars: 500 μm (a, c), 50 μm (b, d).

**FIGURE 6:**
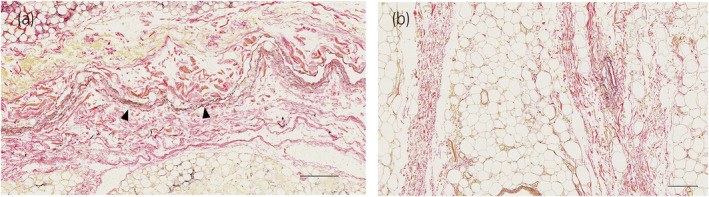
EVG stain. Special microscopic findings of Gf, (a) arrowheads: elastic fibers, (b) Gf present in multiple layers. Scale bars: 200 μm (a, b).

A total of eight kidneys were removed from four cadavers. A total of 36 specimens were prepared from the eight removed kidneys. Out of 36 specimens observed, 26 showed connective tissue that appeared to be Gf. The details are shown in Table 1. Specimens that did not contain Gf were either Gf‐free from the beginning when the large samples were divided into two, or GF layer peeled off when the specimens were sectioned because the layer of Gf is very fragile. In all cadavers, Gf was clearly visible during cross sectioning (Figure 2). Unlike other specimens, specimen No. 26 was prepared by taking a portion of Gf that was identified grossly (Figure 3). The connective tissue in this layer was determined to be mostly collagen fibers, as it stained red with EVG staining (Figure 3b) and blue with MA staining (Figure 3c). The collagen fibers in this layer had a sandwich‐like structure, with a thin layer of thick, long collagen fibers in the central layer, and small granular collagen fibers scattered at the edges, which appears that the collagen fiber bundles are intersecting. Whereas, the epimysium showed densely aligned collagen fibers that were thicker than those of Gf and contained some elastic fibers (Figure 4). Based on the findings in No. 26, we defined the histologic finding of Gf as the collagen‐based connective tissue between the kidney and the fat of the surrounding organs. However, not all of the connective tissue observed as Gf had such a sandwich structure, eight of the 26 specimens had no layered structure and were composed only of small granular collagen fibers (Figure 5). As a special form, specimens No. 2 and 6 contained elastic fibers as part of Gf (Figure 6a). And while most Gf was in a single layer, several specimens were observed as multiple layers of Gf (Figure 6b).

Gf thickness of the 26 specimens averaged 466 μm (SD ±250), and ARCF averaged 27.1% (SD ±10.8). The mean thickness of the epimysium was 754 μm (SD ±67), which tended to be thicker than the Gf, although the difference was not statistically significant. The mean ARCF was 52.8% (SD ±1.1), higher than that of Gf with a statistically significant difference.

## DISCUSSION

Gf is not only an important structure in the classification of renal cancer progression (e.g., Tumor Node Metastasis classification), but also an important marker in renal surgery. Furthermore, in Japan, understanding and proper handling of Gf layer is an important point in the examination for the endoscopic surgery skill certification system approved by the Japanese Society of Endourology and Robotics.^3^, ^4^, ^5^, ^6^ Despite this important structure, there has long been many debates about Gf.^7^, ^8^ In this regard, we think there are two reasons. One is the ambiguity of the definition of Gf. Gf is anatomically described as a membranous structure covering the kidney and the perirenal fat, ureter, adrenal gland, and gonadal veins.^9^, ^10^, ^11^, ^12^, ^13^, ^14^ Another name for Gf is renal fascia, often referring to both the anterior and posterior lobes of the renal fascia. In the last century, the anterior lobe was described as Gf and the posterior lobe as Zuckerkandl fascia, and Gf was sometimes treated as a synonym for anterior lobe of the renal fascia.^8^ Studies by radiologists around the 1980s reported the Gf is vertically longitudinally spindle‐shaped. In these reports, the portion covering the ventral side of the kidney is described as the anterior renal fascia, that between the transversus abdominis fascia and the external colon fascia as the lateroconal fascia, and the two combined and attached to the lumbosacral fascia as the posterior renal fascia.^15^, ^16^, ^17^ This recognition is almost in line with what we urologists recognize when we perform surgery. In kidney surgery, the place where Gf is recognized and detached as a membrane is the so‐called anterior lobe of the renal fascia, and in this study, Gf was defined as the anterior lobe of the renal fascia under consideration.^6^

The second reason is the term “fascia” is not clearly defined. So far, the term “fascia” has been defined variously, including by the Federative International Committee on Anatomical Terminology (1998), the latest British edition of Gray's Anatomy (2008), and the last international Fascia Research Congress.^18^ As an example, Gray's anatomy defines fascia as “a mass of connective tissue of a size visible to the naked eye.” In contrast to the aponeurosis, fascia is described as a “woven” arrangement of fibers.^19^ More recently, a classification based on microscopic molecular mechanics, as opposed to the macroscopic considerations of decades ago, has also been proposed. Based on this new perspective, Huijing and Langevin, in their 2nd Fascia Conference (2009), proposed the inclusion of 12 additional designated terms in the description of fascial tissues. Tissues such as the fascia tendons can be clearly defined by one of these 12 tissue terms.^18^ However, many important areas of the body, including the Gf, are characterized by a gradual transition between such morphological categories, making it difficult to classify and define fascia in a clearly delineated manner. In particular, the visceral fascia to which Gf belongs is thought to be distributed over a large range in terms of the density and regularity of its collagenous tissue. This is consistent with the histological findings of Gf in this study.

With the spread of laparoscopic surgery, it is now possible to perform surgery with a magnified field of view, allowing the surgeon to recognize the minute structures of Gf more than ever before. As a result, several questions have arisen regarding Gf in nephrectomy and other procedures, as noted above. Why does Gf appear to have different structures depending on the surgical approach and the site where Gf is being debrided, despite being the same Gf? We inferred two answers to this question from the histological structure obtained from this study. The first answer is the fragility of Gf. The outer muscular membrane, which was studied as a comparison, is a very strong membrane, and its structure is not easily stretched by external forces. Gf, on the other hand, is composed of very fine‐grained collagen fibers and a very thin layer structure. Therefore, it is a very fragile structure compared to the outer muscular membrane, and it is not difficult to imagine that it can be easily stretched by external forces such as surgical manipulation. It is this fragility that allows surgeons to easily injure the Gf or lose sight of it during dissection. The most important thing for the urologist to realize when performing nephrectomy is the Gf is not always a thick membranous tissue.

The second answer is the diversity of Gf structure. In this study, the basic histological structure of Gf was defined as a sandwich structure, but it was found to have a variety of structures, such as some areas consisting only of small grains of collagen fibers. During surgery, areas with sandwich structures may be easily recognized by the surgeon as membrane. In the present study, Gf tended to be identified as a distinct membrane on the ventral side of the left kidney when specimens were taken. Specimen No. 26, which was clearly recognizable as a membranous structure, was also taken from this site. In addition, this sandwich structure is consistent with the appearance of Gf and fusion fascia when the anterior surface of the kidney is dissected during transabdominal approach surgery, and may be present as the basic structure of Gf at this site. The fact that four of the seven specimens have this structure were collected from the anterior surface of the kidney (the area in contact with the peritoneum and colon) also confirms this. On the other hand, areas composed only of small grains of collagen fibers may be difficult to recognize as membrane. In our surgical experience, in the dissection between the duodenum and the perirenal adipose tissue, Gf is often recognized as a fine foamy structure rather than a membrane. The fact that five of the nine specimens in contact with the duodenum were composed of only small granules of collagen fibers confirms this. Although the small number of specimens does not allow us to draw any conclusions, it is possible that there is a certain regularity in the distribution of Gf structure in this way. In addition, since the present study mainly examined the connective tissue of the ventral organs and the anterior lobe of Gf, we could not examine the area near the upper and lower poles, but the Gfs in this region are very interesting considering that the kidney is a respiratory migratory organ. In addition, whether Gf is monolayered or multilayered may have a significant impact on the appearance of Gf during surgery, and in this study, Gf was observed as multilayered rather than monolayered in 9 of 26 specimens. This variety is probably one of the reasons why Gf appears in a variety of forms rather than uniformly during surgery. The possibility that artifacts from the specimen preparation process are included in the reason for this diversity of observation cannot be ruled out. However, there is no doubt that these are part of the structure of the collagen fibers that make up Gf.

Our primary motivation for conducting this study is to elucidate why Gf appearance differs between transabdominal nephrectomy and retroperitoneal nephrectomy. In transabdominal nephrectomy, Gf can be recognized as a membrane between the ventral organ and the perirenal fat, facilitating the removal of the kidney with Gf wrapping the kidney. In retroperitoneal approach nephrectomy, however, it is often difficult to remove the kidney in Gf‐wrapped state. This question may be explained by the fact Gf is a loose connective tissue. In intraoperative findings, loose connective tissue turns into a membranous structure when fibers are severed from the tissue to which they were attached and no longer have the traction to maintain a three‐dimensional structure of collagen fiber. Thus, when collagen fibers between loose connective tissue are cut, the side with more connective tissue attached is observed as a clear, thick membranous structure, while the side with only a little connective tissue attached is observed as a very thin membrane, which may or may not be recognized. In addition, more connective tissue tends to remain on the far side than on the near side in the direction of the connective tissue peeling, so a firm membranous structure tends to form on the far side than on the near side. In the transperitoneal approach, the far side is the renal side. Therefore, the membranous structures are found on the renal side. In the retroperitoneal approach, the far side is the abdominal side. Therefore, the membranous structures are formed on the peritoneal side. This is not surprising given that Gf is a loose connective tissue composed of very fragile collagen fibers that cannot be divided into a frontal or back surface like a strong membrane that cannot be stretched by external forces, such as the muscular outer membrane.

Finally, a limitation of this study is it is based on a small number of cadavers. Since the condition of connective tissue varies with age, sex, and body size, it must be said that it is difficult to draw firm conclusions from this study. Similar studies should be conducted on a wide variety of cadavers.

## AUTHOR CONTRIBUTIONS

**Kohei Edamura:** Investigation; conceptualization. **Hideyo Ohuchi:** Supervision; conceptualization; investigation; writing – review and editing. **Keita Sato:** Data curation; investigation. **Yasuyuki Kobayashi:** Writing – original draft; conceptualization; investigation; project administration. **Takaaki Komiyama:** Investigation; data curation. **Ryusuke Momota:** Supervision. **Motoo Araki:** Supervision. **Yusuke Tominaga:** Investigation; conceptualization. **Tomoko Kobayashi:** Investigation; conceptualization. **Takehiro Iwata:** Investigation; conceptualization. **Shingo Nishimura:** Investigation; conceptualization. **Takuya Sadahira:** Investigation; conceptualization. **Satoshi Katayama:** Investigation; conceptualization.

## CONFLICT OF INTEREST STATEMENT

Motoo Araki is an Editorial Board member of International Journal of Urology and a co‐author of this article. To minimize bias, they were excluded from all editorial decision‐making related to the acceptance of this article for publication.

## APPROVAL OF THE RESEARCH PROTOCOL BY AN INSTITUTIONAL REVIEWER BOARD

The protocol for this research protocol has been approved by Ethics Committee of Okayama University Graduate School of Medicine, Dentistry and Pharmaceutical Sciences and Okayama University Hospital and it conforms to the provisions of the Declaration of Helsinki (Approval No. 2205‐0033).

## INFORMED CONSENT

Four cadavers were used in this study after obtaining consent from the donors and the bereaved families for the purposes of medical education and research.

## REGISTRY AND THE REGISTRATION NO. OF THE STUDY/TRIAL

N/A.

## ANIMAL STUDIES

N/A.
